# Schetky

**Published:** 1825-03

**Authors:** 


### OBITUARY.

Died lately, at Sierra Leone, —— Schetky, Esq. Deputy Inspector of military hospitals. This gentleman was engaged in active service during the greater part of the Peninsular campaign, and was subsequently attached to the hospital at Fort Pitt. Mr. Schetky was distinguished for the zeal with which he devoted himself to his profession in general, and to the study of morbid anatomy in particular. He possessed the rare and useful accomplishment of drawing with great fidelity and spirit, as may be seen from his delineations of the morbid specimens constituting the pathological collection at Chatham. Many of these are beautifully executed, forming a lasting proof of his industry and acquirements.

